# Relationship between professional experience and nutrition education on nutritional care among surgical nurses

**DOI:** 10.3389/fnut.2026.1866485

**Published:** 2026-08-12

**Authors:** Durdane Yılmaz Güven, Pınar Tunc Tuna

**Affiliations:** 1 Department of Nursing, Faculty of Health Sciences, Karabuk University, Karabuk, Türkiye; 2 Department of Nursing, Akşehir Kadir Yallagöz School of Health, Selçuk University, Konya, Türkiye

**Keywords:** attitudes, knowledge, nurses, nutritional care, perioperative education, surgical nursing

## Abstract

**Background:**

Enhanced Recovery after Surgery (ERAS) protocols highlight the importance of nutritional assessment and support in postoperative care. Nurses are central to implementing these practices, yet knowledge gaps persist. This study examined the relationships between professional experience and nutrition education on nurses’ attitudes, knowledge, and perceived quality of nutritional care in surgical settings.

**Methods:**

A descriptive, correlational study was conducted with nurses (*n* = 133) in surgical and adult intensive care units. Data were collected using a healthcare worker identification form and a validated scale assessing attitudes, knowledge, and perceived quality of nutritional care. Analyses included descriptive statistics, group comparisons, and correlation analyses.

**Results:**

Over half (54.1%) were knowledgeable about the ERAS-related nutritional assessment and support, and 37.6% had received perioperative nutrition training. Mean scores indicated above-average attitudes toward nutritional assessment (23.35 ± 3.97), perceived quality of nutritional care (30.72 ± 8.65), and knowledge of nutritional care (27.87 ± 4.29). Nurses with postgraduate education had higher average attitude and knowledge scores regarding the importance of nutritional assessment compared to others (*p* = 0.01). Nurses who viewed nutrition as an ERAS element in nutritional assessment had higher average attitude scores regarding the importance of nutritional assessment (*p* = 0.01). A weak but significant positive correlation was observed between years of experience in surgical clinics and knowledge scores (r = 0.194, *p* = 0.03).

**Conclusion:**

Surgical nurses demonstrated above-moderate scores in all subdimensions of the Nutrition Care Assessment Scale. Higher educational level, awareness of ERAS-related nutritional assessment and support, and longer surgical nursing experience were associated with greater knowledge and more positive attitudes toward nutritional care.

## 1 Introduction

Enhanced Recovery after Surgery (ERAS) is an evidence-based multidisciplinary approach designed to reduce the physiological stress response to surgery and promote faster postoperative recovery (1). A fundamental component of ERAS is the assessment of patients’ nutritional status and the provision of appropriate nutritional support when indicated, as adequate perioperative nutrition contributes to improved surgical outcomes and recovery (2). Malnutrition is highly prevalent among surgical patients and is associated with increased postoperative complications, prolonged hospital stay, delayed wound healing, and higher morbidity and mortality (3, 4).

Nurses, as the healthcare professionals who maintain the closest and most continuous contact with patients throughout the perioperative period, play a central role in nutritional screening, ongoing nutritional assessment, implementation of nutritional interventions, patient education, and monitoring responses to nutritional therapy (1, 5). Although nurses generally recognize the importance of nutritional care, previous studies have consistently reported deficiencies in evidence-based nutritional knowledge and variations in attitudes and perceived quality of nutritional care among hospital nurses (6–12). These findings suggest that the quality of nutritional care may depend not only on organizational factors but also on nurses’ individual competencies and professional preparation.

Current recommendations for improving evidence-based nutritional care emphasize continuous professional education, standardized clinical protocols, and interdisciplinary collaboration (11, 12). Among these factors, professional experience and nutrition education are considered particularly important because they may influence nurses’ clinical decision-making, confidence, and ability to translate evidence into practice. Previous studies have reported that nurses who receive nutrition-related education generally demonstrate higher levels of nutritional knowledge and more positive attitudes toward nutritional care, whereas increasing clinical experience may strengthen practical skills and clinical judgment in nutritional management (6–14). However, the independent contributions of professional experience and nutrition education to different dimensions of nutritional care have not been comprehensively examined among surgical nurses.

Furthermore, most previous studies have investigated nurses’ nutritional knowledge, attitudes, or perceived quality of care separately rather than evaluating these dimensions simultaneously. Consequently, limited evidence is available regarding how professional experience, nutrition education, and workplace characteristics collectively relate to nurses’ nutritional care competencies in surgical settings. To our knowledge, few studies have concurrently examined the three dimensions of the Theilla et al. (15) Nutritional Care Scale-knowledge, attitudes, and perceived quality-in relation to both professional experience and nutrition education among surgical nurses.

Therefore, this study aimed to examine the relationships between professional experience, nutrition education, and nurses’ attitudes toward nutritional assessment, knowledge regarding nutritional care, and perceived quality of nutritional care among nurses working in surgical wards and surgical intensive care units. By identifying factors associated with evidence-based nutritional care, the findings may contribute to the development of targeted in-service educational strategies and support improvements in perioperative nursing practice.

### 1.1 Research questions

1. What are the scores of nurses working in surgical clinics on the sub-dimensions of the Nutritional Care Assessment Scale (attitude towards the importance of nutritional assessment, perceived quality of nutritional care, and level of knowledge about nutritional care)?
2. Does the nurses’ education level affect their attitude towards the importance of nutritional assessment, perceived quality of nutritional care, and knowledge level regarding nutritional support?
3. Is there a relationship between the length of time working in surgical clinics and the nurses’ attitude towards the importance of nutritional assessment, perceived quality of nutritional care, and knowledge level regarding nutritional support?

## 2 Materials and methods

### 2.1 Design

This descriptive correlational study was conducted to examine the relationships between professional experience, nutrition education, and nurses’ attitudes, knowledge, and perceived quality of nutritional care among nurses working in surgical wards and adult intensive care units.

### 2.2 Population and sample

The study population comprised 195 nurses working in the surgical wards and surgical intensive care units of a training and research hospital between June and December 2024. The required sample size was calculated using the finite population sample size formula with a 95% confidence level and a 5% margin of error, yielding a minimum required sample of 129 participants. The inclusion criteria were as follows: (1) working as a registered nurse in the surgical wards or surgical intensive care units during the study period, (2) having at least six months of clinical experience in the current unit, and (3) voluntarily agreeing to participate in the study. The exclusion criteria were being on maternity leave, sick leave, annual leave, or administrative leave during the data collection period, declining to participate, or returning an incomplete questionnaire. Eligible nurses were recruited using a convenience sampling approach. Data collection was completed after 133 nurses had voluntarily agreed to participate, exceeding the calculated minimum sample size.

### 2.3 Measurement tools

Data were collected using a Healthcare Worker Identification Form and the Questionnaire for Evaluating the Importance of Nutritional Assessment, the Level of Nutritional Care Knowledge and the Perceived Quality of Care among Nurses (15, 16).

*Healthcare Worker Identification Form:* The Healthcare Worker Identification Form was developed by the researchers following a review of the relevant literature (6–8) to collect participants’ sociodemographic characteristics, professional experience, and nutrition-related educational background. The form consisted of three sections and nine questions. The first section collected sociodemographic information, including age, sex, educational level, and marital status. The second section assessed professional characteristics, including years of experience in the current unit, experience in surgical wards, and total nursing experience. The third section gathered information regarding nutrition-related education, including nutritional assessment, nutritional support, and perioperative nutrition education within the ERAS framework. Perioperative nutrition education referred to structured or semi-structured in-service training programs provided within the hospital. These sessions covered the fundamental principles of perioperative nutritional care for surgical patients and were delivered by experienced clinical nurses and/or other healthcare professionals, such as physicians and dietitians. The duration and frequency of the training varied according to the hospital’s in-service education schedule.

*Questionnaire for Evaluating the Importance of Nutritional Assessment, the Level of Nutritional Care Knowledge and the Perceived Quality of Care among Nurses:* The questionnaire was originally developed by Theilla et al. (15) and translated into Turkish and psychometrically validated by Gürlek Kısacık et al. (16). The validated Turkish version of the scale was adopted and used without modification in the present study. The scale consists of 26 items organized into three independently scored subscales. The first subscale assesses nurses’ attitudes toward the importance of nutritional assessment and their perception of nutritional assessment as a fundamental component of nursing care (score range: 7–28). The second subscale evaluates nurses’ knowledge regarding nutritional care (score range: 10–40), whereas the third subscale measures the perceived quality of nutritional care (score range: 9–45). The three subscales were analyzed and interpreted independently. Higher scores indicate more positive attitudes toward nutritional assessment, greater knowledge regarding nutritional care, and higher perceived quality of nutritional care, respectively. In the Turkish validation study, the scale demonstrated satisfactory internal consistency, with an overall Cronbach’s alpha coefficient of 0.80 (16). In the present study, the Cronbach’s alpha coefficients were 0.93 for the Attitudes toward the Importance of Nutritional Assessment subscale, 0.74 for the Knowledge Regarding Nutritional Care subscale, 0.94 for the Perceived Quality of Nutritional Care subscale, and 0.86 for the overall scale.

### 2.4 Data collection

Study data were collected using an online survey method from nurses working in surgical clinics of a training and research hospital who met the inclusion criteria for the study. The research announcement was communicated to nurses through clinical supervisors, and participation was voluntary. Participants were able to access the survey link via mobile devices or computers. Completing the survey form took approximately 10 min on average.

### 2.5 Statistical analysis

Data were analyzed using SPSS version 24. Continuous variables are presented as mean ± standard deviation (SD), along with minimum and maximum values, while categorical variables are presented as frequency (n) and percentage (%). The Shapiro–Wilk and Kolmogorov–Smirnov tests were used to assess the normality of the data. For non-normally distributed variables, comparisons among three or more groups were performed using the Kruskal-Wallis test, and comparisons between two groups were conducted using the Mann–Whitney U test. Correlation analyses were performed to examine relationships between variables. For normally distributed variables, Pearson correlation analysis was applied. In all analyses, *p*-values less than 0.05 were considered statistically significant.

### 2.6 Ethical consideration

Written approvals were obtained from the Karabuk University Non-Interventional Research Ethics Committee (Approval No.: E-77192459-050.99-331739, Date: March 30, 2024), the institution where the study was conducted (Approval No.: E-34771223-774.99-245707159, Date: June 5, 2024), and from the owner of the scale. Informed consent was obtained online from all participants prior to their inclusion in the study.

## 3 Results

A total of 133 nurses participated in the study. Among them, 82.7% were female, with a mean age of 31.56 ± 7.69 years. The majority (69.2%) held a bachelor’s degree. Of the participants, 41.4% worked in intensive care units, with an average of 5.58 ± 5.90 years of experience in surgical wards. Regarding nutritional care, 54.1% of nurses were knowledgeable about the ERAS component of nutritional status assessment and the provision of nutritional support when necessary, while 37.6% had received perioperative nutrition training (Table 1).

**Table 1:** Sociodemographic characteristics, professional experience, and educational status of nurses.

	*n*	(%)
Sociodemographic characteristics		
Gender		
Woman	110	82.7
Male	23	17.3
Marital status		
Married	70	52.6
Single	63	47.4
Educational background		
Licence	92	69.2
Graduate	41	30.8
Age mean ±SD (min - max)	31.56 ± 7.69 (21–52)	
Professional experiences		
The ward where the nurses work		
General surgery ward	21	15.8
Cardiovascular surgery ward	11	8.3
Orthopedic ward	14	10.5
Intensive care units	55	41.4
Emergency room	25	18.8
ENT/Eye/Physical Therapy/Neurosurgery ward	7	5.3
Years of working in surgical services mean ±SD (min - max)	5.58 ± 5.90 (1–25)	
Total working years in the profession mean ±SD (min - max)	8.81 ± 8.26 (1–31)	
Education status		
ERAS element assessment of nutritional status and knowledge of nutritional support if necessary		
There is	72	54.1
None	61	45.9
of receiving education about perioperative nutrition		
There is	50	37.6
None	83	62.7

The mean scores on the scale were as follows: attitudes toward the importance of nutritional assessment, 23.35 ± 3.97; perceived quality of nutritional care, 30.72 ± 8.65; and knowledge of nutritional care, 27.87 ± 4.29. Overall, nurses’ mean scores were above average across all dimensions (Table 2).

**Table 2:** Mean scores of the questionnaire subscales assessing attitudes toward nutritional assessment, knowledge of nutritional care, and perceived quality of nutritional care.

	Mean	Std. deviation	Minimum	Maximum
Attitude towards the importance of nutritional assessment	23.35	3.97	7.00	28.00
Perceived quality of care regarding nutritional care	30.72	8.65	10.00	45.00
Level of knowledge regarding nutritional care	27.87	4.29	10.00	38.00

Comparisons based on educational status revealed that nurses with a postgraduate degree and ERAS knowledge presented higher scores on knowledge of nutritional care than their counterparts. Additionally, nurses with a postgraduate degree demonstrated more positive attitudes toward the importance of nutritional assessment. Nurses who received perioperative nutrition training exhibited higher perceived quality of care scores (*p* < 0.001). Overall, nurses’ mean scores were above average across all dimensions. No statistically significant differences were found in the mean scores for attitudes toward nutritional assessment, perceived quality of nutritional care, and knowledge of nutritional care according to the clinical units in which the nurses worked (*p* > 0.05) (Table 3).

**Table 3:** Comparison of some characteristics and clinics of nurses and scale scores.

Variables	Average attitude score regarding the importance of nutritional assessment	Mean perceived quality of care score for nutritional care	Average score of knowledge level regarding nutritional care
Educational background			
Licence	22.89 ± 3.93	30.26 ± 8.30	27.22 ± 4.50
Graduate	24.37 ± 3.93	31.76 ± 9.40	29.31 ± 3.40
*p*/test statistic	0.01/−2.51*	0.28/−1.09*	0.01/−2.72*
ERAS element assessment of nutritional status and knowledge of nutritional support if necessary			
There is	23.58 ± 4.59	30.95 ± 9.30	28.51 ± 4.40
None	23.07 ± 3.11	30.46 ± 7.89	27.11 ± 4.08
*p*/test statistic	0.08/−1.75*	0.89/−0.138*	0.01/−2.51*
Status of receiving in-service training on perioperative nutrition			
There is	23.70 ± 4.42	33.98 ± 7.93	27.40 ± 5.26
None	23.13 ± 3.69	28.76 ± 8.51	28.15 ± 3.60
*p*/test statistic	0.23/−1.19*	p < 0.001/−3.58*	0.59/−0.54*
Total years of service in the surgical clinic**			
ICU	23.06 ± 4.11	29.88 ± 8.63	27.92 ± 3.99
Surgical Wards	23.75 ± 3.78	31.91 ± 8.62	27.80 ± 4.74
*p*/test statistic	0.41/−0.82	0.33/−0.98	0.92/−0.10

*Mann–Whitney U test **(General surgery, Cardiovascular surgery, Orthopedic, Emergency, ENT/Eye/Physical Therapy/Neurosurgery ward).

Spearman correlation analysis indicated a statistically significant, positive, but weak correlation between years of experience in surgical clinics and total knowledge score (r = 0.020, *p* = 0.03), suggesting that knowledge tends to co-occur with increased clinical experience (Table 4).

**Table 4:** Relationship between nurses’ years of working in surgical clinics and scale scores.

Variables	1	2	3
1. Years working in surgical clinics	—		
2. Total attitude score	r = 0.09 *p* = 0.32	—	
3. Total quality score	r = 0.03 *p* = 0.749	r = 0.14 *p* = 0.11	—
4. Total knowledge score	r = 0.20* p = 0.03	r = 0.11 *p* = 0.20	r = −0.01 *p* = 0.88

*represents the Spearman correlation coefficient.

## 4 Discussion

This descriptive and correlational study examined nurses’ attitudes toward nutritional assessment, knowledge of nutritional care, and perceived quality of nutritional care in relation to professional experience, nutrition education, and knowledge of nutritional assessment and nutritional support as recommended within ERAS guidelines. In this study, 54.1% of participating nurses were knowledgeable about the ERAS component “assessment of nutritional status and provision of nutritional support when necessary,” and 37.6% had received training on perioperative nutrition (Table 1). Previous studies have shown that implementing a standardized postoperative nutrition protocol within the ERAS framework coincides with an earlier transition to oral intake in patients undergoing esophageal resection; however, the prevalence of postoperative malnutrition remained significant (17). Similarly, nutritional supplements provided under the ERAS protocol in patients undergoing liver resection are associated with a shorter length of hospital stay (18). Despite these improvements, the literature emphasizes that nurses’ knowledge of ERAS protocols is generally insufficient. For instance, Güzel and Yava reported that 70.4% of nurses were unfamiliar with ERAS (19), while Ongün and Ak (20) found that 84.5% of nurses lacked awareness of the protocol. Çelebi and İlçe (21) found that 86.8% of nurses had never heard of ERAS protocols. Additionally, Gustafsson et al. (22) reported that healthcare professionals, including nurses, had limited knowledge of ERAS practices in perioperative care. These findings underscore the importance of incorporating the “assessment of nutritional status and provision of nutritional support when necessary” component of the ERAS protocol into nursing education, following current evidence-based approaches. Given the critical role of perioperative nutrition and ERAS in patient recovery, the development and implementation of standardized in-service training programs is recommended to support surgical nurses in adhering to evidence-based guidelines.

In this study, the mean scores of participating nurses were 23.35 ± 3.97 for attitudes toward the importance of nutritional assessment, 30.72 ± 8.65 for perceived quality of nutritional care, and 27.87 ± 4.29 for knowledge of nutritional care (Table 2). Chen et al. (23) reported that nursing support for enteral nutrition can improve the perioperative nutritional status of elderly patients with gastrointestinal tumors by enhancing immune function and promoting intestinal peristalsis. Nurses can systematically identify patients at risk of malnutrition and intervene early using standard screening tools, such as the Subjective Global Assessment or the Malnutrition Universal Screening Tool (24). Similarly, Aliş et al. (6) found that nurses’ mean scores were 24.14 ± 3.13 for attitudes toward nutritional assessment, 24.96 ± 5.67 for knowledge of nutritional care, and 24.18 ± 3.20 for perceived quality of care (5). In a study with intensive care nurses, Kıymaz et al. (9) reported mean scores of 22.7 ± 4.1, 27.0 ± 3.9, and 34.5 ± 6.5 for the same dimensions, respectively (8). The above-average scores observed in all dimensions in the present study are encouraging; however, it is notable that knowledge scores were lower than scores in the other dimensions. This suggests that nurses conceptually recognize the importance of nutritional care and perceive the quality of care as high, yet gaps in evidence-based knowledge persist. Previous studies have similarly highlighted that knowledge of nutritional care within the ERAS framework is not yet widespread among nurses and that structured training programs are needed to improve knowledge (19). Therefore, enhancing nurses’ knowledge is essential to translate their positive attitudes toward nutritional assessment into clinical practice. Considering the critical role of the ERAS protocol and perioperative nutrition in patient recovery, strategies to support continuous professional development in this area are strongly recommended. A noteworthy pattern in the present findings is the discrepancy between nurses’ comparatively lower knowledge scores and their substantially higher perceived quality of nutritional care. This divergence warrants theoretical interpretation rather than uncritical acceptance. Two distinct mechanisms may account for this pattern, and they are not mutually exclusive. First, social desirability bias, the tendency to respond in ways perceived as favorable by others, may have inflated self-reported quality ratings, given that data were collected via self-administered questionnaires in an institutional setting. This is an inherent limitation of cross-sectional, self-report designs and should be interpreted as a measurement constraint rather than a reflection of actual care quality. Second, and conceptually distinct, the observed pattern is consistent with the Dunning-Kruger effect: a metacognitive phenomenon whereby individuals with limited domain-specific knowledge systematically overestimate their competence, precisely because they lack the evaluative framework to recognize their own knowledge gaps (25). In this context, nurses who scored lower on evidence-based knowledge may have assessed their care quality against an internally constructed, rather than externally validated standard of practice. The high attitude scores observed across the sample suggest that nurses genuinely value nutritional care; however, valuing a practice and possessing the evidence-based knowledge to implement it effectively are distinct competencies. This knowledge–perception gap therefore underscores a critical limitation of relying solely on self-assessment for clinical quality monitoring and reinforces the need for objective, competency-based evaluation frameworks in surgical nursing education.

Comparison of nurses’ educational levels and scale scores revealed that nurses with a postgraduate degree and knowledge of ERAS scored higher on knowledge of nutritional care than their counterparts. Similarly, Aliş et al. (6) reported higher knowledge scores among nurses with postgraduate education (5). In another study, Coşğun and Kısacık (7) found that nurses’ knowledge levels were significantly influenced by education (*F* = 9.257; *p* = 0.002) (6). Additionally, nurses with postgraduate education had higher scores for attitudes toward the importance of nutritional assessment, and those who received training on perioperative nutrition achieved higher scores for perceived quality of nutritional care (*p* < 0.001). These findings indicate that education is a key determinant of nurses’ knowledge, attitudes, and perceived quality of care. The study highlights the critical role of structured, up-to-date, and evidence-based educational programs, particularly regarding ERAS and perioperative nutrition, in enhancing professional competence among surgical nurses. This study found no significant differences in nurses’ attitudes towards nutritional assessment, their knowledge levels regarding nutritional care, and their perceived quality of care scores, based on the clinical units in which they worked (*p* > 0.05). This result suggests that knowledge, attitudes, and practices regarding nutritional care may be influenced more by individual and professional characteristics than by the clinical setting. Similarly, Mahmoodpoor et al. (26) reported that the knowledge and attitudes of intensive care nurses regarding nutritional care were particularly related to their education level and professional experience. In addition, Polat Dünya et al. (8) stated that positive attitudes towards nutritional care increase the perceived quality of care and that the level of knowledge affects the perception of the quality of care (7). These findings support the idea that differences in nutritional care can be explained by factors such as education, level of knowledge, and professional development, rather than the clinical field of study. Spearman correlation analysis revealed a statistically significant, positive, but weak correlation between years of experience in surgical clinics and total knowledge score (r = 0.020, *p* = 0.03). This suggests that, while knowledge of nutritional care tends to increase with longer experience, the effect is limited. The weak correlation indicates that experience alone is not sufficient to ensure comprehensive knowledge. The literature presents mixed findings regarding this relationship. Kıymaz et al. reported no significant correlation between years of experience and knowledge scores, whereas Aliş et al. (6) found that nurses with less than one year of professional experience had higher knowledge levels (5, 8). Similarly, Döngel et al. (27) observed that attitude levels varied with professional experience, with nurses having 0–1 year of experience exhibiting higher attitude scores. These differences may reflect the greater exposure of new graduate nurses to ERAS and nutritional care topics within current curricula. Taken together, these results suggest that the relationship of professional experience with knowledge and attitudes is not unidirectional. Knowledge gains do not always parallel the duration of experience, highlighting the importance of continuing professional education in maintaining knowledge levels and facilitating access to current evidence-based practices. The observed correlation between surgical clinical experience and total knowledge scores (r = 0.020) demonstrates that years of routine practice have not fully bridged the gap between acknowledging the ‘importance’ of nutrition and implementing ‘evidence-based’ nutritional care. Furthermore, our data highlight a distinct structural hierarchy. While postgraduate education and direct perioperative training were significantly associated with perceptions of care quality (*p* < 0.001), clinical-level classification did not show a significant distinction. This reality may suggest that the challenges to global ERAS integration stem not from a lack of clinical or basic professional experience, but from a systemic failure to deliver structured, continuous, and dynamic postgraduate metabolic care education.

### 4.1 Limitations

This study has several limitations that should be acknowledged. First, the sample was drawn from a single institution, which may limit the generalizability of the findings to nurses working in different hospitals, regions, or healthcare systems. Therefore, caution should be exercised when interpreting the results beyond the study setting. Second, the cross-sectional and self-reported nature of the data may introduce response bias, as participants’ answers may reflect subjective perceptions rather than objective performance. Thirdly, in the study, the variable of professional experience was evaluated based on the participants’ length of time working in surgical clinics. Therefore, this variable does not fully reflect the total professional experience of the nurses. This should be considered a limitation of the study in terms of the interpretation and generalizability of the findings regarding professional experience. Finally, the study design does not allow causal inferences between professional experience, nutrition education, and nutritional care outcomes.

## 5 Conclusion

The findings showed that surgical nurses had above-moderate scores in all subdimensions of the Nutrition Care Assessment Scale, including attitudes toward the importance of nutritional assessment, perceived quality of nutritional care, and knowledge regarding nutritional care. Nurses with postgraduate education demonstrated significantly higher attitudes toward nutritional assessment and greater knowledge of nutritional care. Similarly, nurses who reported knowledge of nutritional status assessment and nutritional support within the ERAS framework had higher knowledge scores. A weak but significant positive correlation was found between years of experience in surgical clinics and knowledge levels. Overall, educational attainment, ERAS-related knowledge, and professional experience were associated with improved knowledge and more positive perceptions of nutritional care among nurses working in surgical settings.

## Data Availability

The original contributions presented in the study are included in the article/supplementary material, further inquiries can be directed to the corresponding author.
